# Vagus nerve stimulation enhances context discrimination and dentate gyrus neurogenesis through Trk signaling

**DOI:** 10.1093/braincomms/fcag387

**Published:** 2026-10-10

**Authors:** Syed Ahsan Raza, Débora R Calderón-Williams, Navya S Grandhe, Ishayu P Gupta, Christa K McIntyre

**Affiliations:** Department of Neuroscience, University of Texas at Dallas, Richardson, TX 75080, USA; Department of Neuroscience, University of Texas at Dallas, Richardson, TX 75080, USA; Department of Neuroscience, University of Texas at Dallas, Richardson, TX 75080, USA; Department of Neuroscience, University of Texas at Dallas, Richardson, TX 75080, USA; Department of Neuroscience, University of Texas at Dallas, Richardson, TX 75080, USA; Texas Biomedical Devices Center, The University of Texas at Dallas, Richardson, TX 75080, USA

**Keywords:** VNS, fear conditioning, hippocampus, adult hippocampal neurogenesis, BDNF

## Abstract

Adult-born neurons in the hippocampal dentate gyrus support the encoding of distinct representations for similar contexts, a dentate gyrus-dependent function underlying contextual discrimination. Impairment in this function contributes to fear generalization observed in posttraumatic stress disorder. Vagus nerve stimulation is under investigation as a treatment for posttraumatic stress disorder and has been shown to influence hippocampal plasticity, including adult neurogenesis. This raises the question of how vagus nerve stimulation-induced changes in dentate gyrus neurogenesis and plasticity relate to contextual discrimination behaviour. Here, we tested whether vagus nerve stimulation enhances contextual fear discrimination in male and female Sprague–Dawley rats and whether these effects depend on BDNF-TrkB signalling. Rats received 3 days of home cage vagus nerve stimulation using parameters previously shown to enhance memory. Home cage vagus nerve stimulation increased markers of dentate gyrus neurogenesis. The number of Ki67- and doublecortin-expressing cells was greater in the dentate gyrus of vagus nerve stimulation-treated rats than in controls (*P* < 0.005) and (*P* < 0.05), respectively. However, home cage vagus nerve stimulation did not alter contextual discrimination when delivered alone, compared with control treatment (*P* = 0.45). Similarly, vagus nerve stimulation delivered only during conditioning had no behavioural effect (*P* = 0.64). In contrast, combining home cage stimulation with vagus nerve stimulation during conditioning significantly enhanced contextual discrimination between shock-associated and similar contexts (*P* < 0.001). Pharmacological blockade of tyrosine kinase activity at TrkB receptors with K252a prevented both the behavioural enhancement and the vagus nerve stimulation-associated increase in neurogenesis markers. Rats treated with vagus nerve stimulation and vehicle showed better discrimination than rats treated with vagus nerve stimulation and the TrkB inhibitor K252a (*P* < 0.001), and K252a treatment significantly reduced the number of cells expressing Ki67 (*P* < 0.005) and doublecortin (*P* < 0.05) following vagus nerve stimulation. These findings demonstrate that vagus nerve stimulation is associated with BDNF-TrkB signalling-dependent increases in markers of dentate gyrus neurogenesis and enhancement of contextual fear discrimination.

## Graphical Abstract

**Graphical Abstract:**
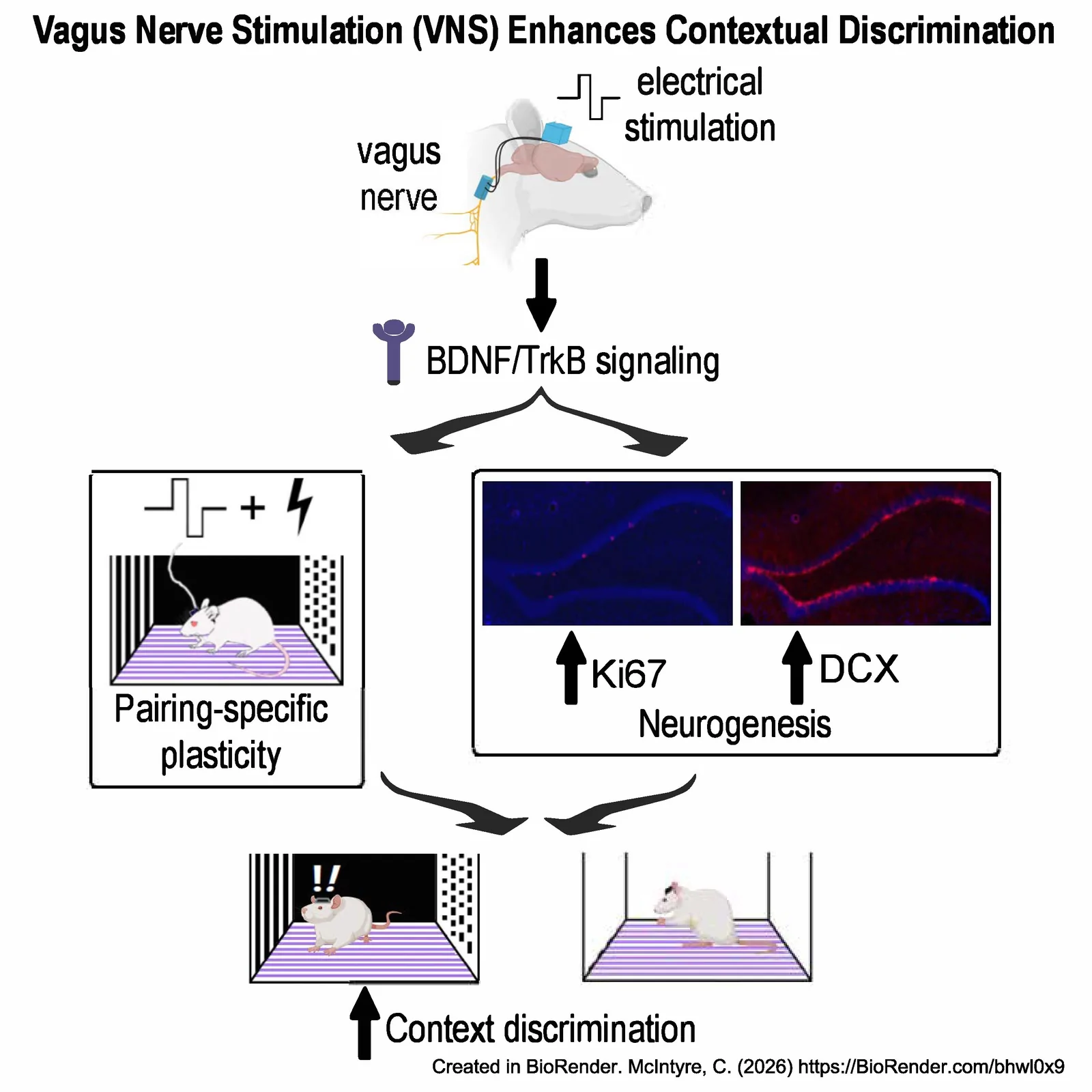

## Teaser

Raza *et al*. discovered that vagus nerve stimulation in the home cage increased markers of neurogenesis in adult rats. Vagus nerve stimulation during fear conditioning significantly improved context discrimination in these rats. However, stimulation in the home cage or during fear conditioning only did not affect context discrimination.

## Introduction

Accurate discrimination between threatening and safe contexts is necessary for everyday survival of organisms. Impairment in this adaptive behaviour contributes to maladaptive fear generalization, which is a hallmark of post-traumatic stress disorder (PTSD) and anxiety disorders.^1,2^ The hippocampal–dentate gyrus (DG) plays a central role in contextual memory formation. The precise balance between pattern separation and pattern completion functions of the DG ensures proper memory formation and later accurate recall and distinguishing between similar contexts.^3-7^ Adult neurogenesis in the DG affects these functions, facilitating fine distinctions between overlapping contextual representations, thereby reducing inappropriate fear responses.^8-10^ Thus, treatments that enhance DG neurogenesis hold promise for improving fear discrimination and reducing pathological fear.^11^

Vagus nerve stimulation (VNS) is an FDA-approved treatment for epilepsy, depression, and stroke. Based on preclinical evidence showing that pairing VNS with conditioned cues enhances extinction of conditioned fear memory,^12-14^ it is currently under investigation as an adjuvant to exposure therapy for PTSD.^15^ Unpaired VNS increases cell proliferation in the DG,^16-18^ and increases expression of brain-derived neurotrophic factor (BDNF) in the hippocampus.^17,19,20^ BDNF regulates neuroplasticity and adult neurogenesis,^21-23^ and BDNF-TrkB signalling supports the integration and survival of newborn neurons in the DG, making it a likely mediator of VNS-induced effects on neurogenesis. However, whether these unpaired VNS effects influence contextual discrimination is not known. In this study, we examined whether VNS facilitates contextual discrimination in an experience- and TrkB-dependent manner. Using a contextual fear discrimination (CFD) paradigm, we compared the effects of VNS delivered in the home cage, VNS paired with conditioning, and the combination of both manipulations. In parallel, we quantified markers of DG neurogenesis to determine whether VNS is associated with changes in proliferative and immature neuronal populations. Finally, we tested the necessity of BDNF-TrkB signalling using systemic administration of the TrkB antagonist K252a.

## Methods

### Animals

All animal experiments were conducted according to the NIH guidelines for the care and use of laboratory animals and approved by the Institutional Animal Care and Use Committee (IACUC) at The University of Texas at Dallas (protocol #2023-0121). In this study, 94 male and female Sprague–Dawley rats (Taconic Biosciences, USA), approximately 3 months old and weighing 280–300 g at the start of experiments were used. Rats were acclimatized to the vivarium for 1 week before surgical implantation of VNS electrodes and then singly housed. Each rat was handled for 5 min per day for 3 consecutive days before receiving VNS or sham stimulation in the home cage. Rats were kept on a 12:12 h light–dark cycle with ad libitum access to food and water. All experiments were conducted during the light phase.

### Vagus nerve cuff electrode implant

VNS cuff electrodes were constructed in our laboratory,^24^ and the surgical implantation procedure has been previously described.^13^ Briefly, animals were anaesthetized with isoflurane (2%–3% in oxygen, Western Medical Supply, CA, USA). A 5 mm incision was made on the scalp, and a 2 cm ventral midline incision was made above the tip of the sternum. A subcutaneous tunnel was created from the neck incision to the top of the head for the insulated wires connecting the VNS cuff electrode to a headcap. The neck muscles (sternomastoid, sternohyoid and omohyoid) were bluntly dissected and retracted to expose the left cervical vagus nerve adjacent to the carotid artery. The cuff electrode was positioned around the vagus nerve and sutured closed. To confirm proper functioning of the cuff electrode, a brief stimulation (0.8 mA, 20 Hz, 5 s) induced the Hering–Breuer reflex, a momentary cessation of breathing.^25^ The muscle retractors were then removed, and the neck incision was sutured. While still under anaesthesia, the rat was placed on a stereotaxic frame, and a headcap containing a 4-pin connector was secured to the skull using acrylic and bone screws. Each rat received subcutaneous injections of antibiotic (Baytril; 10 mg/kg) and anti-inflammatory (Rimadyl; 5 mg/kg) medications, as well as 5 ml normal saline during surgery. Sham rats underwent the same surgical procedures, except that the vagus nerve was not stimulated in any experiment. All rats were allowed to recover from surgery for at least 1 week.

### Vagus nerve stimulation

To test the effect of VNS on hippocampal neurogenesis in rats, VNS was administered using parameters that are effective for enhancing memory.^26^ Stimulation of the vagus nerve through the cuff electrode was delivered with an isolated pulse stimulator (Model 2100, A-M Systems, USA). A commutator connected the VNS stimulating cables from a controller board to the headcap on the rat and a custom MATLAB programme triggered stimulation. Each rat received five stimulations (30 s, 30 Hz, 100 µs pulse width, 0.8 mA, inter-stimulus interval 300 s) per session, one session per day for 3 consecutive days in a fresh bedding cage designated as the home cage (Fig. 1A). The Sham group was attached to the VNS cable but did not receive stimulation. Three days after the home cage VNS, rats were transcardially perfused with 1× phosphate-buffered saline (PBS) followed by a 4% paraformaldehyde (PFA) solution and brains were flash frozen in ice-cold 2-methylbutane for immunohistochemistry.

**Figure 1:**
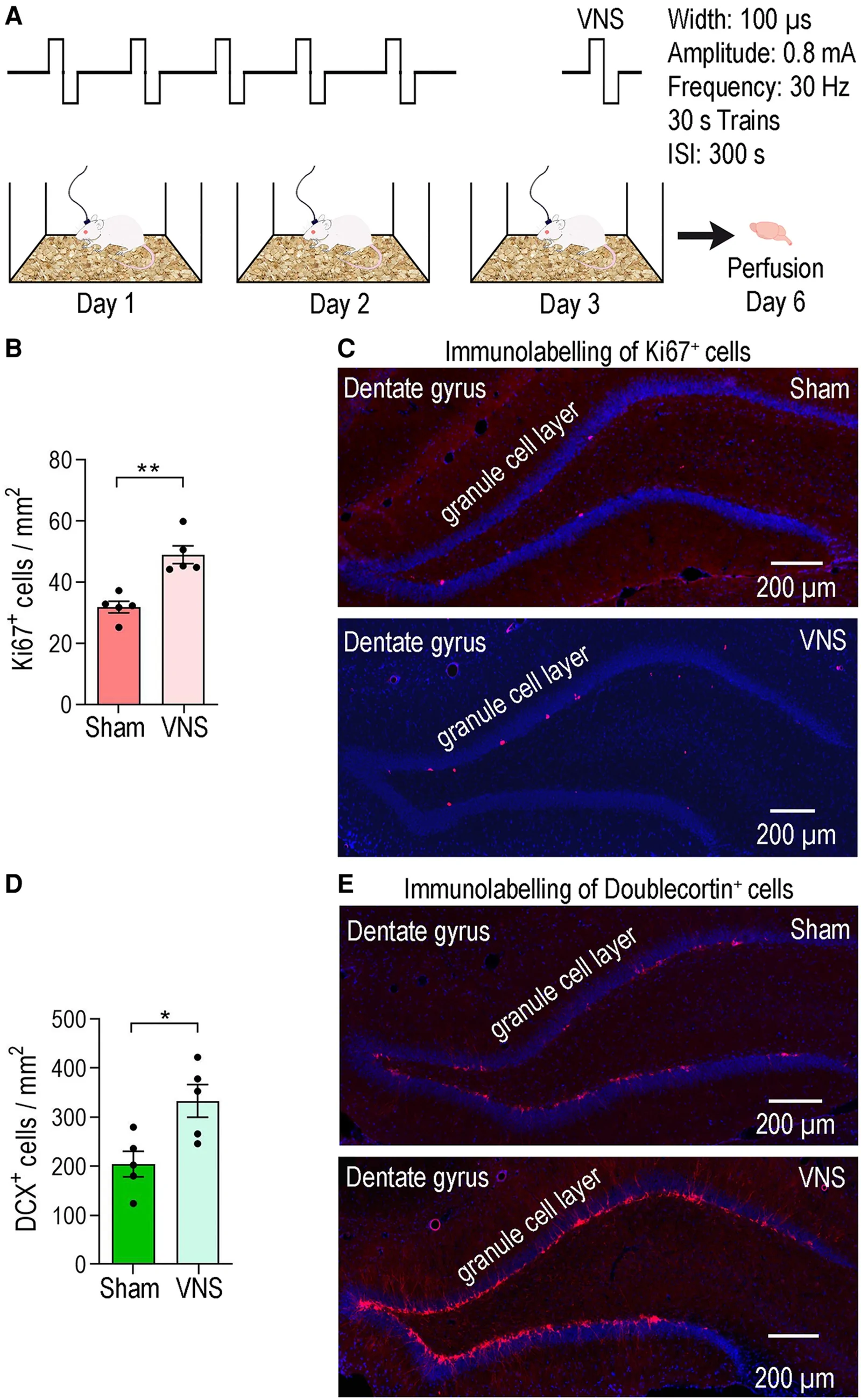
**Vagus nerve stimulation (VNS) delivered in the home cage promotes adult hippocampal neurogenesis.** (**A**) Schematic of the home cage VNS regimen. Rats received five VNS bouts per day for three consecutive days, and brains were collected 3 days after the final stimulation session. (**B)** Quantification of Ki67^+^ cells in the DG. VNS significantly increased Ki67^+^ cell number compared to sham group (*n* = 2 males and *n* = 3 females, each). (**C**) Representative microscopic images showing DAPI nuclear stain (blue) and Ki67^+^ immunolabelling (red) in the DG (upper: VNS, lower: Sham). Scale bar 200 µm. (**D**) Quantification of doublecortin-positive (DCX^+^) cells in the DG. VNS significantly increased DCX^+^ cell number compared to sham group (*n* = 2 males and *n* = 3 females, each). (**E**) Representative microscopic images showing DAPI (blue) and DCX^+^ (red) labelling in the DG (upper: VNS, lower: Sham). Scale bar 200 µm. Data are presented as mean ± SEM. Statistical analysis was performed using Student’s unpaired *t*-test. *P < 0.05; **P < 0.01.

### Contextual fear discrimination task

To test the effect of VNS on CFD, rats learned to discriminate between a fearful shock context and a similar (non-shock) context in a 5-day-long non-randomized CFD paradigm.^10,27^ One week after the final home cage stimulation, rats were placed in the shock context that consisted of a Plexiglas booth (31 × 23 × 21 cm) with metal bar flooring for footshock delivery, housed inside a soundproof behaviour box (61 × 82 × 57 cm) equipped with a ceiling light and a camera (Logitech). The walls were covered with black and white laminated patterns (Fig. 2A), and a 2% acetic acid solution served as the contextual odour. Before the rat was placed in the shock context, the VNS cable was attached to the headcap. After 2 min, a 30 s VNS train was initiated. A 0.6 mA, 1 s footshock was delivered in the middle of each VNS train. Each rat received five VNS-shock pairings and remained in the chamber for 90 s after the final stimulation. Sham animals had the VNS cable attached and received five 1 s footshocks, but VNS was not triggered. Four hours later, rats were placed in a similar context for 6 min. The similar context had transparent Plexiglas walls, the same metal bar flooring, a 30% ethanol odour, and did not include shocks or the VNS cable. Rats experienced both contexts in this way for five consecutive days. CFD testing was conducted 48 h later. Rats were tested (no shock, no VNS) for 6 min each in the shock context first, and testing was conducted in the similar context 4 h later.

**Figure 2:**
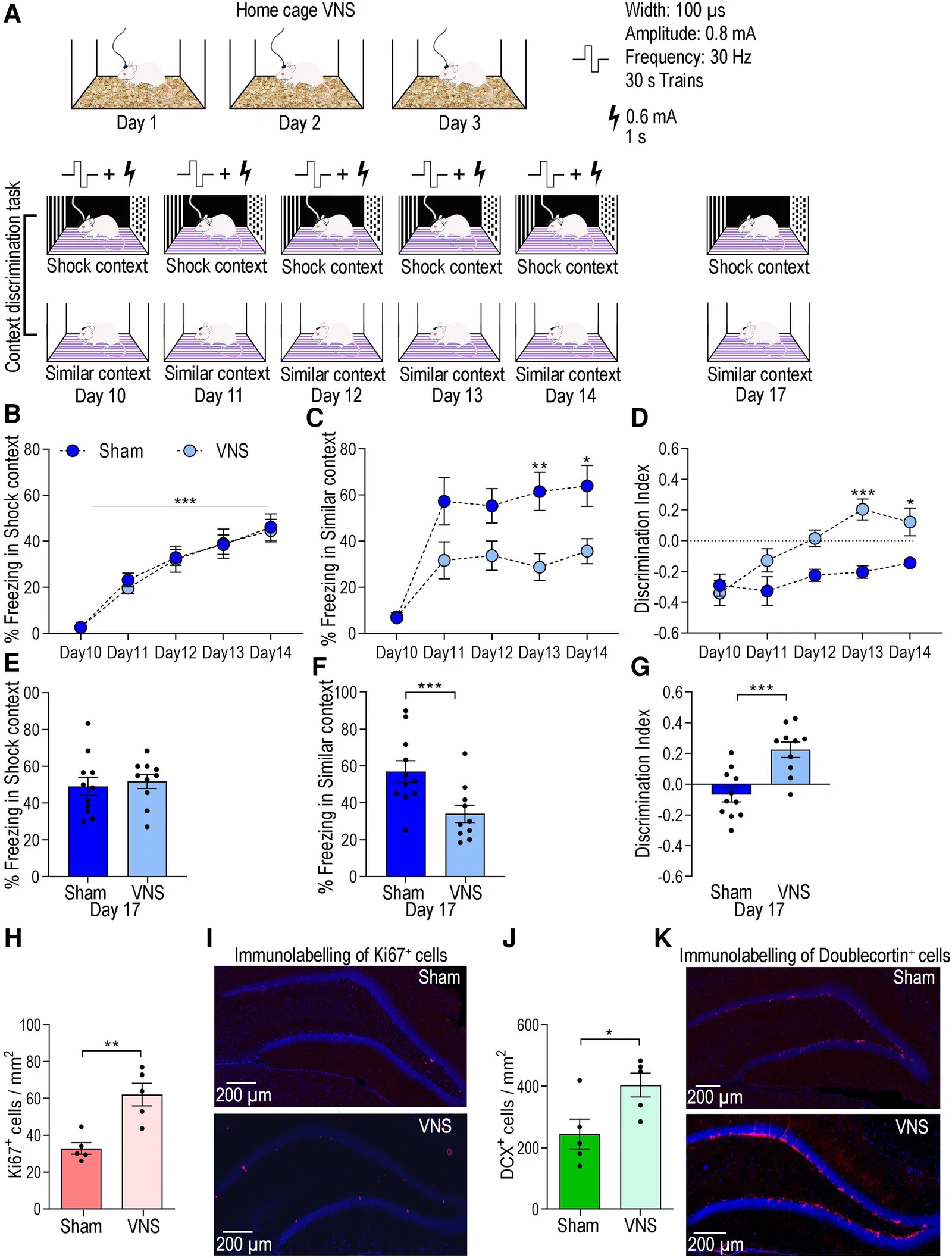
**Vagus nerve stimulation enhances contextual fear discrimination.** (**A**) Schematics of the home cage VNS regimen and the contextual fear discrimination (CFD) task. One week after home cage VNS, rats received five VNS-footshock pairings in the shock context and were exposed to the similar context without stimulation 4 h later. This procedure was repeated for five consecutive days. CFD testing was conducted on Day 17 by exposing rats to the shock context followed by the similar context, both without stimulation. (**B**) Freezing in the shock context does not differ between the VNS (*n* = 5 males and *n* = 5 females) and Sham groups (*n* = 6 males and *n* = 5 females) during CFD training. (**C**) In the similar context, VNS-treated rats exhibit significantly reduced freezing compared to Sham on days 13 and 14. (**D**) The discrimination index is significantly higher in the VNS compared to Sham group on days 13 and 14. (**E**) On the CFD test Day 17, freezing in the shock context does not differ between groups. (**F**) VNS-treated rats exhibit significantly reduced freezing in the similar context on the test day. (**G**) The discrimination index on the test day is significantly greater in the VNS group. (**H**) Quantification of Ki67^+^ cells in the DG shows a significant increase in the VNS-treated rats compared with Sham controls (*n* = 2 males and *n* = 3 females, each). (**I**) Representative microscopic images showing DAPI (blue) and Ki67^+^ (red) immunolabelling in the DG (upper: VNS, lower: Sham). Scale bar 200 µm. (**J**) Quantification of DCX^+^ cells in the DG shows a significant increase in the VNS-treated rats compared with Sham controls (*n* = 2 males and *n* = 3 females, each). (**K**) Representative microscopic images showing DAPI (blue) and DCX^+^ (red) immunolabelling in the DG (upper: VNS, lower: Sham). Scale bar 200 µm. Data are presented as mean ± SEM. Statistical analysis was performed using two-way repeated-measures ANOVA followed by Bonferroni *post hoc* test in (**B–D**), and Student’s unpaired *t*-test in (**E–J**). *P < 0.05; **P < 0.01; ***P < 0.001.

Rats were perfused transcardially with 1× PBS followed by 4% PFA after CFD testing. Brains were flash frozen for immunohistochemistry. Freezing behaviour, defined as more than 1 s of complete immobility aside from breathing or ear twitching, was video recorded and scored by two experimenters who were blind to treatment group assignments. Freezing score was calculated as the percentage of time spent freezing in the first minute of the training session, while the first 2 min were analysed for the recall sessions. The discrimination index for each rat was calculated as (% freezing in shock context − % freezing in similar context)/(% freezing in shock context + % freezing in similar context). To test whether BDNF contributes to VNS-driven modulation of CFD, we used K252a (a non-specific tyrosine kinase inhibitor with activity at TrkB receptors) to block the TrkB pathway. K252a was dissolved in saline/DMSO mixture and injected intraperitoneally at 25 µg/kg.^28^ Injections were given 45 min before each home cage stimulation and each shock context session. A saline/DMSO injection served as Vehicle control.

### Immunohistochemistry

Rabbit anti-Ki67 (Abcam, ab16667-1001) at a dilution of 1:250 and rabbit anti-DCX (Abcam: ab207175) at a dilution of 1:100 were used as primary antibodies. As a secondary antibody, biotinylated goat anti-rabbit (Vector Labs, ba-1000-1.5) at a dilution of 1:200 was used and visualized through conjugation with streptavidin Cy5 (Jackson Immuno, 016170084) at a dilution of 1:1000. Sections were mounted with Vectashield containing 4′,6-diamidino-2-phenylindole (DAPI; Vector Laboratories H1200) and imaged using a slide scanner (Evident Scientific VS200).

For quantification of Ki67^+^ (cell proliferation) and DCX^+^ (immature neurons), brains were serially cut into 40-µm-thick coronal sections. Six sections per animal were stained, and immunopositive cells were counted in a blinded manner through the anterior–posterior extent of the granule cell layer in the DG. Area sizes were measured with CellSens software, and cell densities were expressed as positive cells per square millimetre. Mean cell densities from six sections per rat were calculated and used for group comparisons.

### Statistical analysis

All values are given in group means ± SEM. Normality of data was tested with the Shapiro–Wilk test. Comparison of two groups was conducted with Student’s two-tailed unpaired *t*-test. For multiple group comparisons, two-way analysis of variance (ANOVA) followed by Bonferroni *post hoc* test was used. No animal or data points were excluded from the analysis. All rats that completed the experimental procedures and perfusion were included in the final datasets. The data analysis and graph creation were conducted with GraphPad Prism. Differences were considered statistically significant if *P* < 0.05.

## Results

### Vagus nerve stimulation in the home cage promotes adult hippocampal neurogenesis

To determine whether home cage VNS alters markers of adult neurogenesis in the DG, rats received five VNS bouts per day (frequency: 30 Hz, Width: 100 µs, amplitude: 0.8 mA, ISI: 300 s), each lasting 30 s, for three consecutive days. Sham animals underwent identical handling and procedures without stimulation. Rats were perfused 2 days after the final stimulation, and DG tissue was analysed for proliferating cells (Ki67^+^) and immature neurons (DCX^+^) using immunohistochemistry (Fig. 1A). Home cage VNS significantly increased the number of Ki67^+^ cells in the DG compared with Sham controls (*t*(8) = 4.84, *P* = 0.001, Fig. 1B and C), as well as the number of DCX^+^ cells (*t*(8) = 3.02, *P* = 0.01, Fig. 1D and E).

### Vagus nerve stimulation facilitates contextual fear discrimination

We next examined whether VNS alters CFD. To address this question, we used a non-randomized CFD task in which rats learn to discriminate a shock-associated context from a similar, non-shock context across repeated exposures (Fig. 2A).^10,27^ Rats received VNS or Sham treatment in their home cages for 3 days, followed by 5 days of fear conditioning in the shock context. No VNS was delivered in the similar context.

During training, freezing behaviour in the shock context did not differ between groups (Fig. 2B). A two-way repeated-measures ANOVA revealed significant main effects of time (*F*(4,95) = 33.05, *P* < 0.001), but no main effect of treatment (*F*(1,95) = 0.17, *P* = 0.682) and no treatment × time interaction (*F*(4,95) = 0.07, *P* = 0.990), indicating that VNS did not alter fear expression in the shock-associated context. In contrast, freezing behaviour in the similar context differed between groups (Fig. 2C). A two-way repeated-measures ANOVA revealed significant main effects of treatment (*F*(1,95) = 23.30, *P* < 0.001) and time (*F*(4,95) = 12.41, *P* < 0.001), with no significant treatment × time interaction (*F*(4,95) = 1.69, *P* = 0.158). *Post hoc* comparison showed that VNS-treated rats exhibited significantly reduced freezing compared with Sham controls on Day 13 (*P* = 0.007) and Day 14 (*P* = 0.028), consistent with improved discrimination of the similar, non-shock context. Analysis of the discrimination index further supported this interpretation (Fig. 2D). A two-way repeated-measures ANOVA revealed significant main effects of treatment (*F*(1,95) = 24.65, *P* < 0.001) and time (*F*(4,95) = 8.26, *P* < 0.001), as well as a significant treatment × time interaction (*F*(4,95) = 3.06, *P* = 0.02). *Post hoc* comparison indicated higher discrimination indices in VNS-treated rats on Day 13 (*P* < 0.001) and Day 14 (*P* = 0.03), reflecting a progressive enhancement of contextual discrimination across training. During the retention test on Day 17, freezing in the shock context did not differ between groups (*t*(19) = 0.41, *P* = 0.68, Fig. 2E). However, VNS-treated rats exhibited significantly reduced freezing in the similar context (*t*(19) = 2.99, *P* = 0.007, Fig. 2F) and significantly higher discrimination indices overall (*t*(19) = 4.28, *P* < 0.001, Fig. 2G). One-sample *t*-tests showed that discrimination index on Day 17 was significantly above chance (0) in VNS-treated rats (*t*(9) = 4.51, *P* = 0.001), whereas Sham rats did not differ from chance (*t*(10) = 1.45, *P* = 0.176). Consistent with the effects observed in the initial neurogenesis experiment, rats that received VNS during CFD training showed significantly higher numbers of Ki67^+^ cells (*t*(8) = 4.20, *P* < 0.003, Fig. 2H and I) and DCX^+^ cells (*t*(8) = 2.57, *P* < 0.03, Fig. 2J and K) in the DG compared with Sham controls. Collectively, these results show that VNS increases markers of DG neurogenesis and enhances CFD without altering freezing in the shock context.

### VNS in the home cage or during conditioning alone does not affect contextual fear discrimination

To determine whether enhanced CFD requires VNS delivered in the home cage, during conditioning, or the combination of both, these manipulations were tested separately. First, rats received home cage VNS or Sham treatment for 3 days and then completed the CFD paradigm without VNS during conditioning (Fig. 3A). In the shock context (Fig. 3B), a two-way repeated-measures ANOVA revealed a significant main effect of time (*F*(5,96) = 13.88, *P* < 0.001), but no main effect of treatment (*F*(1,96) = 0.005, *P* = 0.944) and no treatment × time interaction (*F*(5,96) = 0.06, *P* = 0.997). Similarly, in the similar context (Fig. 3C), there was a significant main effect of time (*F*(5,96) = 7.36, *P* < 0.001), but no main effect of treatment (*F*(1,96) = 0.16, *P* = 0.68), and no treatment × time interaction (*F*(5,96) = 0.20, *P* = 0.96). Analysis of the discrimination index (Fig. 3D) showed a significant main effect of time (*F*(5,96) = 4.36, *P* = 0.001), but no effect of treatment (*F*(1,96) = 0.55, *P* = 0.45), and no treatment × time interaction (*F*(5,96) = 0.39, *P* = 0.85). During the retention test on Day 17, freezing in the shock context (*t*(16) = 0.87, *P* = 0.39, Fig. 3E), similar context (*t*(16) = 0.60, *P* = 0.55, Fig. 3F) as well as discrimination index (*t*(16) = 0.76, *P* = 0.45, Fig. 3G) did not differ between groups. One-sample *t*-tests indicated that discrimination index on Day 17 did not differ from chance (0) in either group (Sham: *t*(8) = 1.77, *P* = 0.113; VNS: *t*(8) = 0.32, *P* = 0.755). Together, these results indicate that home cage VNS alone does not alter freezing behaviour or contextual discrimination.

**Figure 3:**
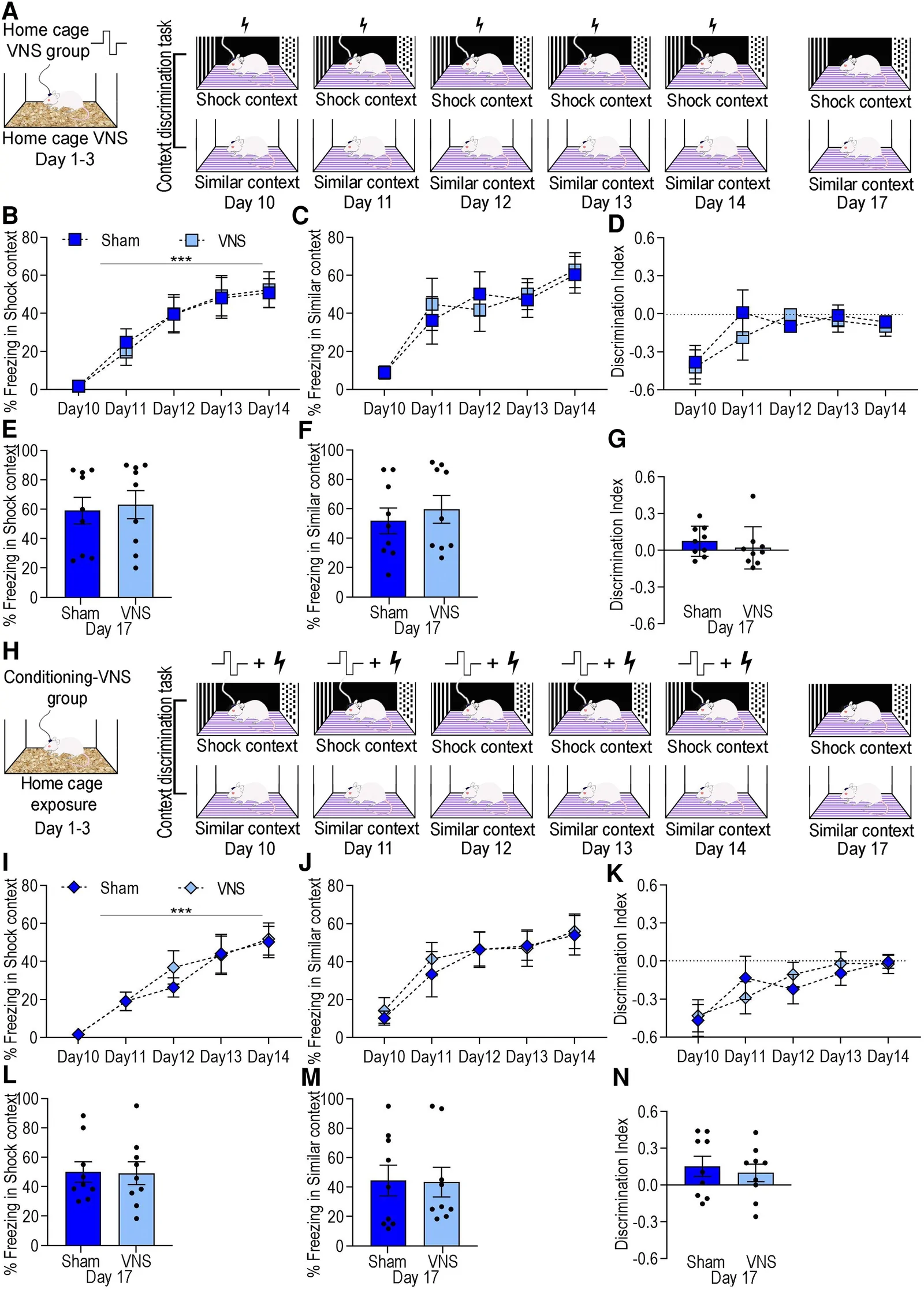
**Vagus nerve stimulation delivered in the home cage or during the shock context conditioning alone does not affect contextual fear discrimination.** (**A**) Schematic of the experimental design in which rats receive VNS in the home cage and no VNS is delivered during conditioning in the contextual fear discrimination (CFD) task. Rats were plugged into the stimulator but they did not receive VNS during conditioning. (**B**) Home cage VNS alone does not alter freezing in the shock context, nor (**C**) in the similar context during the CFD test. (**D**) The discrimination index does not differ between VNS (*n* = 5 males and *n* = 4 females) and Sham (*n* = 5 males and *n* = 4 females) groups. On CFD test Day 17, freezing in the (**E**) shock context and (**F**) similar context does not differ between groups, and (**G**) the discrimination index remains unchanged. (**H**) Schematic of the experimental design in which rats receive VNS only during conditioning in the CFD task. Rats were plugged in but they did not receive VNS in the home cage. (**I**) VNS delivered during conditioning does not alter freezing in the shock context, or (**J**) similar context during the CFD test. (**K**) The discrimination index does not differ between the VNS (*n* = 5 males and *n* = 4 females) and Sham (*n* = 5 males and *n* = 4 females) groups. On CFD test Day 17, freezing in the (**L**) shock context and (**M**) similar context does not differ between groups, and (**N**) the discrimination index remains unchanged. Data are presented as mean ± SEM. Statistical analysis was performed using two-way repeated-measures ANOVA followed by Bonferroni *post hoc* test in **B–D** and **I–K**, and Student’s unpaired *t*-test in **E–G** and **L–N**. ***P < 0.001.

We next examined whether VNS delivered exclusively during conditioning, in the absence of prior home cage stimulation, affected CFD performance (Fig. 3H). In the shock context (Fig. 3I), a two-way repeated-measures ANOVA revealed a significant main effect of time (*F*(5,96) = 15.12, *P* < 0.001), but no main effect of treatment (*F*(1,96) = 0.17, *P* = 0.67) and no treatment × time interaction (*F*(5,96) = 0.192, *P* = 0.96). In the similar context (Fig. 3J), time again had a significant effect (*F*(5,96) = 5.35, *P* < 0.001), whereas neither treatment (*F*(1,96) = 0.14, *P* = 0.71) nor the treatment × time interaction (*F*(5,96) = 0.07, *P* = 0.99) was significant. Analysis of the discrimination index (Fig. 3K) showed a significant main effect of time (*F*(5,96) = 6.79, *P* < 0.001), but no treatment effect (*F*(1,96) = 0.0001, *P* = 0.99), and no treatment × time interaction (*F*(5,96) = 0.42, *P* = 0.83). During the retention test on Day 17, freezing in the shock context (*t*(16) = 0.08, *P* = 0.93, Fig. 3L), similar context (*t*(16) = 0.07, *P* = 0.94, Fig. 3M) as well as discrimination index (*t*(16) = 0.47, *P* = 0.64, Fig. 3N) did not differ between groups. One-sample *t*-tests indicated that discrimination index on Day 17 did not differ from chance (0) in either group (Sham: *t*(8) = 1.83, *P* = 0.103; VNS: *t*(8) = 1.38, *P* = 0.203).

Together, these results demonstrate that neither home cage VNS alone nor VNS delivered exclusively during conditioning is sufficient to alter freezing behaviour or enhance CFD.

### Blockade of BDNF signalling prevents VNS-dependent enhancement of contextual fear discrimination

BDNF signalling is necessary for adult hippocampal neurogenesis,^21,22,29^ and VNS influences both neurogenesis and BDNF expression in the hippocampus.^19,20^ To determine whether BDNF-TrkB signalling is required for the behavioural benefits of VNS, TrkB receptor activation was blocked using K252a (a tyrosine kinase inhibitor with activity at TrkB). Rats received intraperitoneal injections of either K252a (25 µg/kg) or Vehicle 45 min before each home cage VNS session and before each conditioning session, and all rats received VNS during both phases (Fig. 4A).

**Figure 4:**
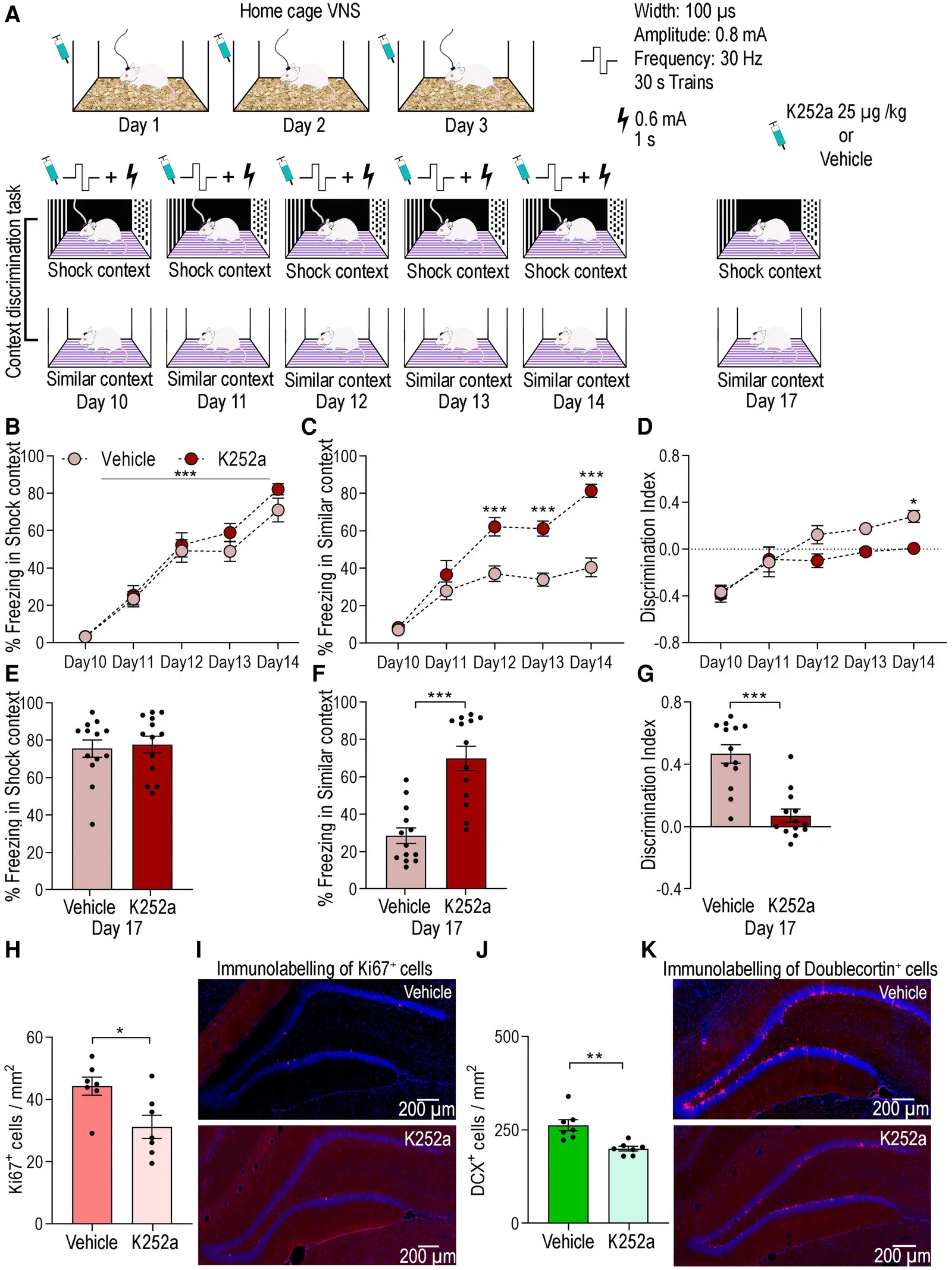
**Blockade of BDNF-TrkB signalling prevents VNS-dependent enhancement of contextual fear discrimination.** (**A**) Schematic of the experimental paradigm. Rats received intraperitoneal injections of K252a (25 µg/kg) or Vehicle 45 min before each home cage VNS session and before each conditioning session during CFD. Both groups received VNS throughout the experiment. (**B**) Freezing in the shock context does not differ between the K252a group (*n* = 7 males and *n* = 6 females) and the Vehicle group (*n* = 7 males and *n* = 6 females) during CFD training. (**C**) In the similar context, K252a-treated rats exhibit significantly increased freezing compared to Vehicle-treated controls across training days. (**D**) The discrimination index is significantly lower in the K252a group compared to the Vehicle group during training. (**E**) On the CFD test Day 17, freezing in the shock context does not differ between groups. (**F**) In the similar context, K252a-treated rats exhibit significantly higher freezing compared to Vehicle-treated controls on the test day. (**G**) The discrimination index on the test day is significantly lower in the K252a group. (**H**) Quantification of Ki67^+^ cells in the DG shows a significant increase in the VNS-treated rats compared with Sham controls (*n* = 4 males and *n* = 3 females, each). (**I**) Representative microscopic images showing DAPI (blue) and Ki67^+^ (red) immunolabelling in the DG (upper: VNS, lower: Sham). Scale bar 200 µm. (**J**) Quantification of DCX^+^ cells in the DG shows a significant increase in the VNS-treated rats compared with Sham controls (*n* = 4 males and *n* = 3 females, each). (**K**) Representative microscopic images showing DAPI (blue) and DCX^+^ (red) immunolabelling in the DG (upper: VNS, lower: Sham). Scale bar 200 µm. Data are presented as mean ± SEM. Statistical analysis was performed using two-way repeated-measures ANOVA followed by Bonferroni *post hoc* test in (**B–D**) and Student’s unpaired *t*-test in (**E–J**). *P < 0.05; **P < 0.01; ***P < 0.001.

During training, freezing behaviour in the shock context did not differ between groups (Fig. 4B). A two-way repeated-measures ANOVA revealed a significant main effect of time (*F*(4,12) = 71.97, *P* < 0.001), but no main treatment effect (*F*(1,12) = 3.16, *P* = 0.07) and no treatment × time interaction (*F*(4,12) = 0.55, *P* = 0.69). In contrast, freezing behaviour in the similar context differed between groups (Fig. 4C). A two-way repeated-measures ANOVA revealed significant main effects of time (*F*(4,12) = 44.63, *P* < 0.001) and treatment (*F*(1,12) = 56.28, *P* < 0.001), as well as significant time × treatment interaction (*F*(4,12) = 6.55, *P* < 0.001). *Post hoc* analysis revealed that K252a-treated rats exhibited significantly greater freezing than vehicle-treated controls on Day 12 (*P* < 0.001), Day 13 (*P* < 0.001), and Day 14 (*P* < 0.001). Analysis of the discrimination index (Fig. 4D) revealed significant main effects of time (*F*(4,12) = 16.50, *P* < 0.001) and treatment (*F*(1,12) = 9.48, *P* < 0.003). *Post hoc* comparisons showed that Vehicle-treated rats exhibited significantly greater discrimination than K252a-treated rats on Day 14 (*P* = 0.03). During the testing session on Day 17, freezing in the shock context did not differ between groups (*t*(24) = 0.33, *P* = 0.73, Fig. 4E). However, K252a-treated rats exhibited significantly greater freezing in the similar context (*t*(24) = 5.35, *P* < 0.001, Fig. 4F) and a significantly lower discrimination index (*t*(24) = 5.49, *P* < 0.001, Fig. 4G). One-sample *t*-tests further showed that Day 17 discrimination index was significantly above chance (0) in vehicle-treated rats (*t*(12) = 7.94, *P* < 0.0001), whereas K252a-treated rats did not differ from chance (*t*(12) = 1.67, *P* = 0.12). Consistent with these behavioural effects, K252a treatment significantly reduced the number of Ki67^+^ (*t*(12) = 2.76, *P* = 0.01, Fig. 4H and I) and DCX^+^ cells (*t*(12) = 3.86, *P* = 0.002, Fig. 4J and K) in the DG compared to Vehicle-treated controls, showing that blockade of TrkB signalling prevented the VNS-associated increase in DG neurogenesis markers.

## Discussion

In this study, we show that VNS engages hippocampal mechanisms that support CFD in an experience-dependent manner. Specifically, home cage VNS in behaviourally naïve rats increased markers of adult hippocampal neurogenesis, but this manipulation alone was not sufficient to enhance contextual discrimination. Behavioural facilitation emerged only when VNS during contextual discrimination learning was preceded by home cage stimulation, indicating that both prior and task-relevant stimulation are required. Importantly, pharmacological blockade of BDNF-TrkB signalling prevented both the VNS-associated increase in neurogenesis and the enhancement of CFD. Together, these findings indicate that VNS enhances contextual discrimination only under conditions in which stimulation is combined with behavioural experience. These effects occurred alongside increases in markers of DG neurogenesis, suggesting that, in addition to previously reported effects of VNS on synaptic plasticity such as hippocampal LTP, neurogenesis-related processes may also contribute to its cognitive effects.

Recent work implicates noradrenergic modulation of the DG in contextual disambiguation. Tonic elevations of norepinephrine within the DG enhance discrimination between similar contexts,^29^ pharmacological enhancement of noradrenergic signalling during learning improves episodic memory specificity weeks later while preserving reactivation of dorsal hippocampal engram cells in rats.^30^ Other recent findings suggest that LC activation predicts temporal pattern separation in DG activity.^31^ VNS activates vagal afferents that project to the nucleus tractus solitarius and subsequently engages the locus coeruleus (LC),^32^ increasing noradrenergic signalling in downstream targets, including the hippocampus. Prior work showed that optogenetic inhibition of LC neurons interfered with VNS enhancement of extinction memory,^33^ suggesting a potential contribution of LC-derived norepinephrine to VNS-induced memory modulation. The LC provides dense innervations to the DG,^34^ and high-frequency stimulation produces long-term potentiation (LTP) at perforant path–DG synapses in an adrenergic-dependent manner *in vitro*^35,36^ and *in vivo*.^37,38^ Orexin-driven modulation of the LC similarly produces long-lasting potentiation of DG synapses.^39^ Consistent with these findings, previous studies report that VNS can promote LTP in the DG.^40^

VNS has also been shown to induce widespread upregulation of BDNF, possibly through norepinephrine signalling,^41^ generating conditions that support neuronal survival, synaptic remodelling and long-term plasticity.^19^ In the DG, VNS increases progenitor proliferation and the number of immature neurons.^16,17^ The present study extends these findings by demonstrating VNS-dependent increases in neurogenesis markers (Fig. 1). These findings are consistent with the possibility that VNS promotes a permissive state in the DG that involves neurotrophic signalling and changes in progenitor proliferation and may support synaptic potentiation and hippocampal function.^17,18,42^ One such function particularly sensitive to these forms of plasticity is pattern separation, the DG-mediated transformation of highly similar inputs into distinct neuronal representations.^3,4^ This computational function is critical for discrimination learning, especially in tasks requiring the differentiation of overlapping contexts. Mounting evidence demonstrates that neurogenesis in the DG contributes substantially to this function, as adult-born granule cells are preferentially recruited during tasks that demand fine contextual discrimination and are essential for reducing memory interference.^8-10^ Conversely, reduced DG neurogenesis is linked to increased generalization of fear memories and deficits in discrimination learning, emphasizing the link between DG circuitry and adaptive memory processing.^43^

VNS is being actively explored as an adjunctive intervention for fear- and anxiety-related disorders in both rodents^13^ and humans.^15^ Our previous work has shown that VNS enhances extinction of conditioned fear when paired with presentation of the conditioned stimulus in the absence of the unconditioned stimulus, and that it prevents reinstatement and renewal of conditioned fear in rat models of PTSD.^13,14^ Administration of VNS during extinction training enhances plasticity in the pathway between the infralimbic prefrontal cortex and the basolateral amygdala, a critical circuit involved in extinction memory.^12^ Consistent with these preclinical findings, the first in-human study using direct VNS during exposure therapy was recently published and reported clinically meaningful improvements in PTSD symptoms.^15^ Whether VNS could also enhance discrimination between similar contexts, however, had not been addressed. The present study fills this gap by demonstrating that pairing VNS with learning in the shock context facilitates discrimination performance, enabling rats to more effectively distinguish a shock-associated context from a perceptually similar safe context (Fig. 2).

A plausible interpretation of our findings is that VNS engages two complementary processes in the DG. Home cage VNS increases markers of proliferation and immature granule cells, and may thereby expand or alter the state of the population of adult-born neurons present during CFD, including cells entering a phase of heightened plasticity. VNS paired with conditioning could then act on this primed population, together with established neuromodulatory effects of VNS^29^ to facilitate DG computations that support discrimination between similar contexts. Recent work shows that norepinephrine signalling in the DG can enhance contextual discrimination and salience processing,^29^ raising the possibility that LC-mediated neuromodulation contributes to the behavioural effects of VNS. Consistent with this view, facilitation of contextual discrimination occurred only when VNS was administered both in the home cage and during conditioning, as neither manipulation alone altered freezing behaviour or discrimination performance (Fig. 3). Although home cage VNS increased neurogenesis, it did not improve discrimination in the absence of behavioural pairing. This aligns with previous studies showing that VNS must be temporally paired with training to be effective.^44^ Together, these findings are consistent with a framework in which home cage stimulation alters the cellular state of the DG, while VNS paired with learning engages plasticity mechanisms during encoding that support discrimination learning.

Pharmacological manipulation in this study shows that systemic administration of K252a, a non-specific tyrosine kinase inhibitor, prevents both the VNS-induced increase in neurogenesis and the associated improvement in discrimination performance. Although the present findings do not isolate TrkB-specific or DG-restricted mechanisms, they are consistent with a model in which VNS-driven LC neuromodulation and tyrosine kinase-dependent signalling in DG converge to support the plasticity required for enhanced context discrimination. In line with previous work showing that K252a pre-treatment blocks VNS-induced TrkB phosphorylation in the hippocampus,^45^ our data indicate that tyrosine kinase signalling is required for the behavioural effects of conditioning-paired VNS paradigm and are consistent with a critical contribution of BDNF-TrkB signalling. An additional observation from this study is that VNS did not enhance memory for the aversive context itself. At first glance, this may appear inconsistent with previous reports that VNS can strengthen learning and memory,^46,47^ including contextual fear memory.^48,49^ However, in our task both Sham and VNS groups acquired robust fear to the training context, suggesting that the emotional salience of the shock was sufficient to drive strong associative learning in all animals. Experimental groups did not differ in how strongly they learned the aversive context, instead, VNS accurately improved how the rats differentiated it from a similar environment. This improvement aligns with a role for VNS in refining contextual coding rather than amplifying fear acquisition.

This study provides initial evidence that VNS-associated neurogenesis contributes to improvements in context discrimination learning. Several limitations should be considered and addressed in future studies. Ki67^+^ and DCX^+^ immunolabeling identify proliferating cells and immature neurons, respectively, but these markers do not fully resolve the different stages of the adult neurogenic lineage or establish the long-term survival and functional integration of newly generated neurons. Moreover, Ki67 and DCX were quantified only in the home cage VNS and combined home cage plus VNS paired with fear conditioning conditions, so changes in neurogenesis in the conditioning-only cohorts remain unknown. Future studies incorporating additional lineage markers (e.g. GFAP/SOX2 for neural progenitors) or thymidine analogue labelling approaches such as BrdU^16^ would allow direct tracking of neuronal maturation and persistence throughout the CFD paradigm. The causal association between adult-born neurons and discrimination behaviour should be studied in future research using selective ablation, optogenetic manipulation, or activity tagging to test whether VNS-induced neurogenesis is required for the behavioural effects observed here. Because we did not label newly generated neurons at defined time points or selectively manipulate adult-born neurons, our two-stage model should be regarded as hypothesis-generating rather than a mechanism demonstrated by our data. Although our interpretation focuses on BDNF-TrkB signalling, we did not quantify BDNF in this cohort; its role is inferred from prior VNS studies rather than shown directly in our animals. Finally, VNS engages multiple neuromodulatory systems, and interactions with cholinergic or serotonergic pathways may also shape discrimination learning.

In conclusion, our study demonstrates that VNS enhances contextual discrimination when stimulation is paired with learning and that these behavioural effects require intact BDNF-TrkB signalling. VNS was also associated with increases in markers of DG neurogenesis. Together, these findings outline a cellular and behavioural framework through which VNS may influence DG-dependent processes relevant to pattern separation and hippocampal functions, and highlight its potential as a therapeutic approach for improving fear discrimination.

## Data Availability

The data supporting the results are available from the corresponding author upon reasonable request.
